# Optimized generation of high-resolution phantom images using cGAN: Application to quantification of Ki67 breast cancer images

**DOI:** 10.1371/journal.pone.0196846

**Published:** 2018-05-09

**Authors:** Caglar Senaras, Muhammad Khalid Khan Niazi, Berkman Sahiner, Michael P. Pennell, Gary Tozbikian, Gerard Lozanski, Metin N. Gurcan

**Affiliations:** 1 Center for Biomedical Informatics, Wake Forest School of Medicine, Winston-Salem, North Carolina, United States of America; 2 Office of Science and Engineering Laboratories, Center for Devices and Radiological Health, Food and Drug Administration, Silver Spring, Maryland, United States of America; 3 Division of Biostatistics, College of Public Health, The Ohio State University, Columbus, Ohio, United States of America; 4 Department of Pathology, The Ohio State University, Columbus, Ohio, United States of America; University of Michigan, UNITED STATES

## Abstract

In pathology, Immunohistochemical staining (IHC) of tissue sections is regularly used to diagnose and grade malignant tumors. Typically, IHC stain interpretation is rendered by a trained pathologist using a manual method, which consists of counting each positively- and negatively-stained cell under a microscope. The manual enumeration suffers from poor reproducibility even in the hands of expert pathologists. To facilitate this process, we propose a novel method to create artificial datasets with the known ground truth which allows us to analyze the recall, precision, accuracy, and intra- and inter-observer variability in a systematic manner, enabling us to compare different computer analysis approaches. Our method employs a conditional Generative Adversarial Network that uses a database of Ki67 stained tissues of breast cancer patients to generate synthetic digital slides. Our experiments show that synthetic images are indistinguishable from real images. Six readers (three pathologists and three image analysts) tried to differentiate 15 real from 15 synthetic images and the probability that the average reader would be able to correctly classify an image as synthetic or real more than 50% of the time was only 44.7%.

## Introduction

In clinical practice, Immunohistochemistry (IHC) is widely used to localize specific epitopes of molecules in cells and tissues that aid in diagnosis and prognosis of cancer [1–3]. IHC also plays a critical role in selecting a proper systemic therapy for cancer patients [2]. Generally, IHC markers are used according to specific guidelines where the intensity of stains and the number of positively stained cells are expressed as a percentage of all malignant cells. In clinical practice, IHC stain interpretation is often carried out manually. The prediction consists of counting each positively- and negatively-stained malignant cell under a microscope [4] and calculating the ratio of positive to total malignant cells. Faced with this daunting task and shortage of time, some pathologists revert to visual estimation of this ratio [3]. As expected, the manual ratio estimation suffers from poor reproducibility even in the hands of expert pathologists [2, 5, 6].

A traditional approach for the evaluation of computerized quantitative image analysis methods includes having an expert diligently generate a reference standard (e.g., by segmenting structures or by counting cells), and then comparing the computer results to the reference standard. However, due to inter- and intra-observer variability in performing a quantitative task on digital pathology images [7], a reference standard generated by one expert is often considered inadequate, and multiple experts’ interpretation is sought. Involving multiple experts results in a resource-intensive evaluation process and limits the sample size for the evaluation. If the ground truth were known, as in the case of synthetically generated images, the effort for the evaluation would be immensely reduced, and much larger evaluation data sets could be used, reducing the uncertainty inherent due to limited sample sizes.

There have been some efforts to develop synthetic histopathological images. Cheikh et al. recently developed a synthetic histological image generation algorithm by modeling tumor architecture and spatial interactions in breast cancer [8]. Although the statistical properties of the generated synthetic images (i.e., the number of tumor patterns, their shape and their area) were similar to those of real images, the models created ‘unrealistic’ details in the synthetic images. In a recent study by our group [9], we manually generated a collection by extracting a group of Ki-67 positive and negative nuclei from images of Ki-67 stained follicular lymphoma biopsies. Our algorithm generated synthetic tissue sections with known percentages of positive and negative nuclei by using this collection. Although the statistical characteristics of the nuclei and their appearance mimicked real cases, the visual variance of the nuclei was dependent on the richness of the created collection, and the tissue background appeared unrealistic. As a result, neither of these approaches could create realistic images to match pathologists’ expectations or to validate analytical methods.

In this study, we developed a novel approach for creating synthetic digital histopathological slides by artificial neural networks. In recent years, the convolutional neural networks (CNN) have become a critical workhorse for many different image processing problems [10–12]. A novel application of the CNN is in Generative Adversarial Networks (GAN) with a goal to “make the output indistinguishable from reality” [13]. Our method is a variation of a GAN, termed conditional GAN (cGAN), which allows generating very realistic histopathological images with fully controlled ground truth [14]. We believe that an important application area for our synthetic IHC image generation method is the evaluation of quantitative image analysis methods for IHC slides. By using our method, we can generate realistic looking positive and negative nuclei with different shape, size, and spatial distributions.

## Method

### Dataset collection

In this study, we collected Ki67-stained whole slide images from 32 different breast cancer patients. This study is IRB approved by the OSU Cancer Institutional Review Board (OSU-15136), Office of Responsible Research Practices, with Waiver of Consent Process, and Full of Waiver of HIPAA Research Authorization. For this particular application, we scanned these slides using an Aperio ScanScope (Leica Biosystems Inc., Buffalo Grove, IL) at a 40x magnification where the pixel size is 0.2461 x 0.2461 μm^2^. An experienced breast pathologist carefully annotated these slides for tumor and non-tumor regions. We randomly selected a total of 84 region-of-interest (ROI) images within the tumor region. Each ROI has a size of 2300x1200 pixels which is equivalent to one high-power-field. We intentionally selected this size to provide the pathologists with the similar environment when they analyze a slide at 40x magnification under a microscope. We experimented with two different input data types to train our system: 1) user annotations mask and 2) segmentation output mask.

After the input data generation, all of the ROIs were divided into tiles of size 256x256 pixels. Any tile that doesn’t contain a positive or a negative nucleus was excluded from the dataset. There were a total of 694 tiles, 572 of which were used for training, and the remaining 122 for visual validation. We followed two different approaches to train our system.

#### User annotation mask

To create the training dataset, all of the stain-positive and stain-negative nuclei in the ROIs were marked manually. A stain-positive (or negative) nucleus means that a cell within a tissue is stained positively (or negatively). To ensure the quality of the annotations, we worked with four trained operators. Each operator first annotated the entire positive and negative nuclei with colored dots in 21 ROIs, analyzed the annotations of another operator, and corrected any annotation errors. No area, orientation or shape information was recorded because the nuclei were represented by only coordinate information represented by dots.

#### Computer segmentation mask

**Fig 1:**
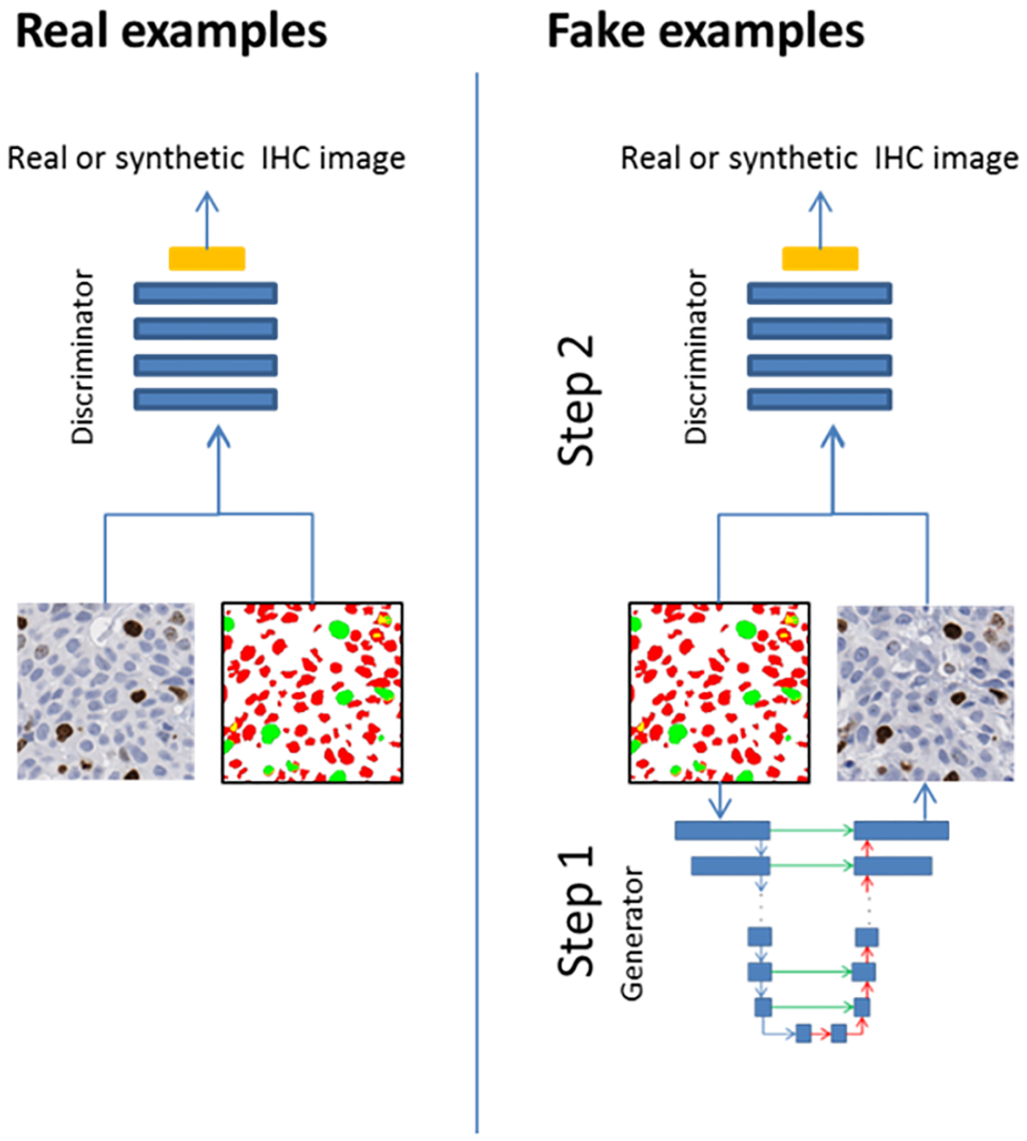
Training of discriminator network. *For real examples*, *we used the real images and their segmentation/annotation masks (M*^*i*^, $I_{r}^{i}$*) as an input. The* green and red colored annotations correspond to Ki67 positive and Ki67 negative nuclei, respectively. *For fake examples*, *we applied a two-step procedure*. *In Step 1*, *we used generator (U-net) algorithm to create a synthetic image by using the segmentation/annotation*. *In Step 2*, *the output of the generator and initial segmentation (M*^*i*^, $I_{g}^{i}$*) are used as an input for D*.

As a second approach, we trained our system with the output of a nuclei segmentation technique that we developed in a prior study [5]. To illustrate the process, in Fig 1, the colors green and red represent the regions that are segmented as Ki67 positive and Ki67 negative, respectively by our technique. The yellow color was used for lightly stained positive regions, which may occur as staining artifacts or background staining. For each tile, we generated the nuclei segmentation and used it as an input for our cGAN neural network similar to the implementation in [13].

### Phantom image generation

As mentioned earlier, a cGAN is computational model to generate realistic looking synthetic images. It consists of two main components: a generator (**G**) and a discriminator (**D**). During the training, the generator learns to produce realistic looking images without a prior knowledge of underlying probability distribution. Simultaneously, the discriminator learns to distinguish between real images and the images produced by the generator. The main idea is to devise a system where synthetic images produced by the generator become indistinguishable from real images. The technical details necessary to implement our method are described below.

For a given real image, $I_{r}^{i}$, let *M*^*i*^ represent its corresponding user annotations or segmentation output mask. The generator **G**, tries to create output images, $I_{g}^{i}$, that cannot be distinguished by **D** from real images. The final objective function, *L*_*final*_ is defined as:

$$L_{final}=\text{arg}\underset{G}{\;\text{min}}\underset{D}{\;\text{max}}L_{cGAN}(G,D)+L_{l1}(G)$$

where L_*cGAN*_(*G*, *D*) is part of the objective function which **D** tries to maximize while learning on how to distinguish real pairs (*M*^*i*^, $I_{r}^{i}$) from fake pairs (*M*^*i*^, $I_{g}^{i}$). Simultaneously, **G** tries to minimize L_*cGAN*_(*G*, *D*) and synthesize fake images that would deceive D. Here, L_l1_(G) is the difference of output $I_{g}^{i}$, and the ground truth, $I_{r}^{i}$, as L1 distance [13].

**Fig 2:**
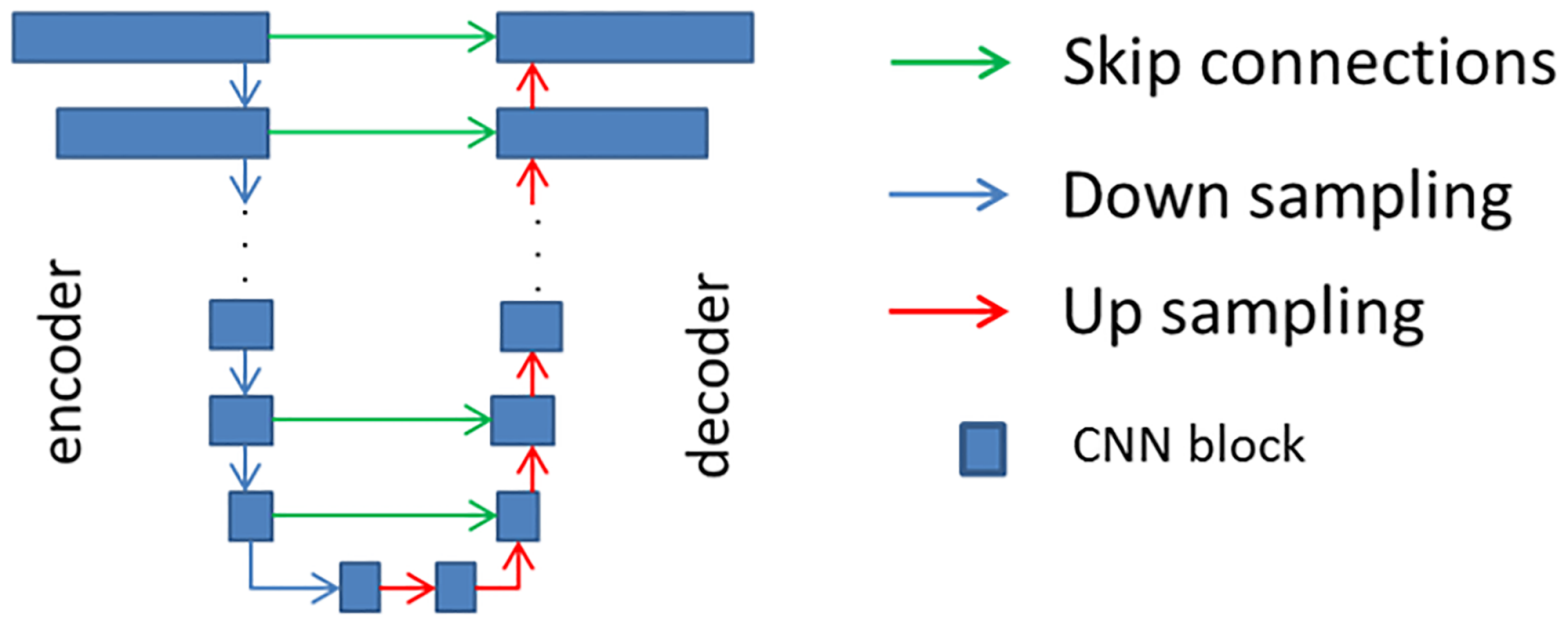
Used neural network framework for generator, G.

In the study, as a generator, we used a modified version of the “U-net” [15], whose architectural overview is shown in Fig 2. All CNN blocks described in Fig 2 includes 3x3 CNNs with 2x2 strides, Batch Normalization [16], and leak Relu layers [17]. The generator includes 16 CNN blocks, eight of those are used for encoding and the remaining eight are used for decoding. For larger images, the number of blocks may be increased. The number of the filters at i^th^ CNN block, n_i_, is defined as:

$$n_{i}=64*2^{\text{min}(3,\;L-0.5-|i-L-0.5|)}$$

where L is the number of layers in the encoder and decoder, and is equal to eight in the current setup.

As discriminator, **D**, we used a CNN based classifier “patchGAN” [13]. The classifier includes four CNN blocks and a convolution layer with a 1-dimensional output. The image is divided into small tiles and for each patch; patchGAN tries to identify the input as real or fake. The final output is the average of all responses.

During the training of the proposed method, we followed the standard approach [13, 18], such that one gradient descent step on **D** is followed by one gradient descent step on **G** for optimization. The training procedure for **D** is given in Fig 1. The network is optimized with Adam [19] for 200 epochs with the batch size of four.

**Fig 3:**
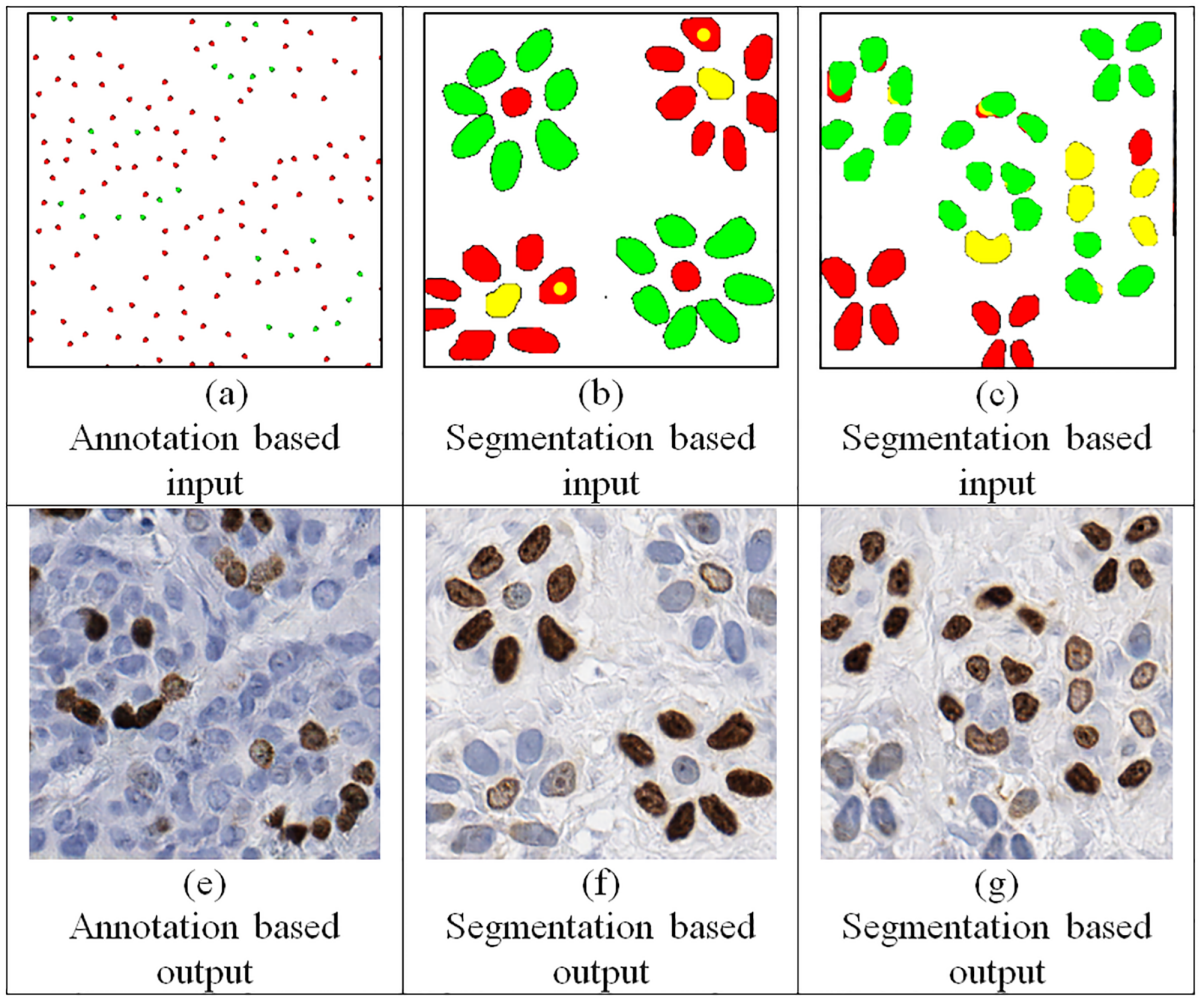
Fully synthetic images (e-g). We created several toy data to generate synthetic images with different characteristics by using annotation based input (a) and segmentation based input (b and c).

For inference, the method provides the freedom to manually create a scenario which allows: 1) defining different spatial overlap of nuclei, 2) placement of different sized nuclei at certain locations, and 3) control over spatial frequency of nuclei during synthetic data generation (Fig 3). We fed the input data to G and skipped the D to create an inferred synthetic image, i.e. we do not use **D** during the inference. Besides since the G does not include fully connected layers, it is possible to generate larger images during the inference.

#### Experiments

We trained our method for both of the datasets (i.e. “user annotated”, and “segmentation output masks”) separately. We trained two systems with different input types, and the comparison of their results is presented in the results section. We tested our algorithm on an independent set of 122 randomly selected validation images, none of which were used during the training. We used each $I_{r}^{i}$, and it’s corresponding *M*^*i*^ to create a synthetic image with the same characteristics as $I_{r}^{i}$.

In our first experiment, we worked with three image analysts and three pathologists for their visual evaluations. To maintain the attention of the observers, we divided the experiment into three parts. In each part, we showed a dataset of 10 images and asked the observers to identify synthetic images. To make the parts unbiased, the distributions of the synthetic images in the three datasets were kept confidential. The dataset for the first part included 10 synthetic images. The second dataset included 10 real images, and final dataset included five real and five synthetic images.

Reader accuracy in identifying the correct image type (real versus synthetic) was analyzed using a hierarchical Bayesian logistic regression model containing random effects for images and readers. The random reader effects accounted for heterogeneity in reader accuracy while the random image effects accounted for heterogeneity in the difficulty of images. Diffuse or non-informative priors were assigned to all parameters, and the posterior inference was obtained using Metropolis-Hastings sampling run for 500,000 iterations following a 5,000-iteration burn-in. Sampled values were used to calculate the posterior probability that the average reader would be able to identify the correct image type more than 50% of the time if presented with an image of average difficulty. Two readers (Image Analysts 1 and 2) were excluded from this analysis since we did not record their decisions on individual images; we just tabulated the number correct and incorrect for each data set. As a secondary analysis, the hierarchical model was extended to included fixed effects of data set to determine if performance differed by ratio of real to artificial cases. Modeling was performed using PROC MCMC in SAS Version 9.4 (SAS Inc, Cary, NC).

**Fig 4:**
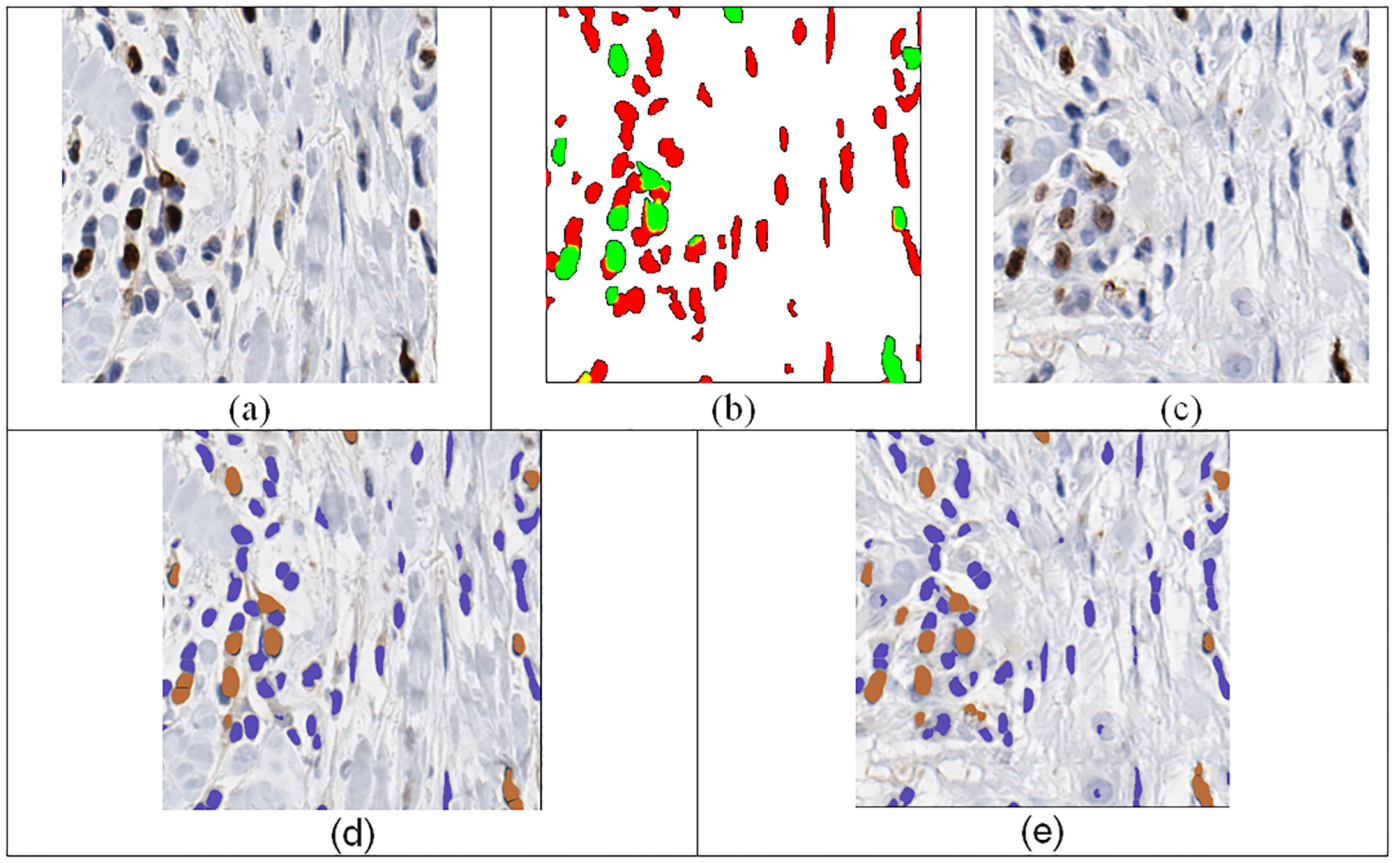
Example (a) real image, (b) segmentation result based on [5], (c) synthetic image used for evaluation of computerized quantitative method, (d) visual ImmunoRatio output for the real image, visual ImmunoRatio output for synthetic image.

As a prerequisite to the claim that images generated by our technique can produce images that can be used for evaluation of computerized quantitative methods, we demonstrated that quantitative methods perform similarly for real and synthetic images. To test our method in a situation similar to the example in Fig 3, we used a real data set of 122 Ki-67 stained images that was completely independent from the cGAN training data set. We call this data set as the *quantitative comparison dataset*. For each image patch in our quantitative comparison data set, we aimed at generating a synthetic image that is different from the real image in terms of its overall appearance (i.e., location and spatial arrangement of the cells) but is similar to the real image in terms of Ki-67 quantitation. To achieve this, we used a segmentation algorithm that was previously developed in our laboratory [5] to generate a segmentation mask, and applied the segmentation mask and the real image as the input to the cGAN. We used the output of the cGAN as the synthetic image. An example of the real and synthetic images used in this experiment is shown in Fig 4.

If the cGAN output is suitable for the evaluation of computerized quantitative methods, then a quantitative method applied to the real and cGAN-generated images should provide similar results, as discussed above. To test this, we applied a quantification method that uses a fundamentally different segmentation algorithm from our segmentation algorithm to both real and synthetic images. The quantification method, ImmunoRatio, calculates the percentage of positively stained nuclear area by using a color deconvolution algorithm for separating the staining components and adaptive thresholding for nuclear area segmentation [20]. Agreement between ImmunoRatio values measured on real images and their artificial replicas was quantified using Lin’s concordance correlation [21] and visualized using a Bland-Altman plot [22].

## Results and discussion

**Fig 5:**
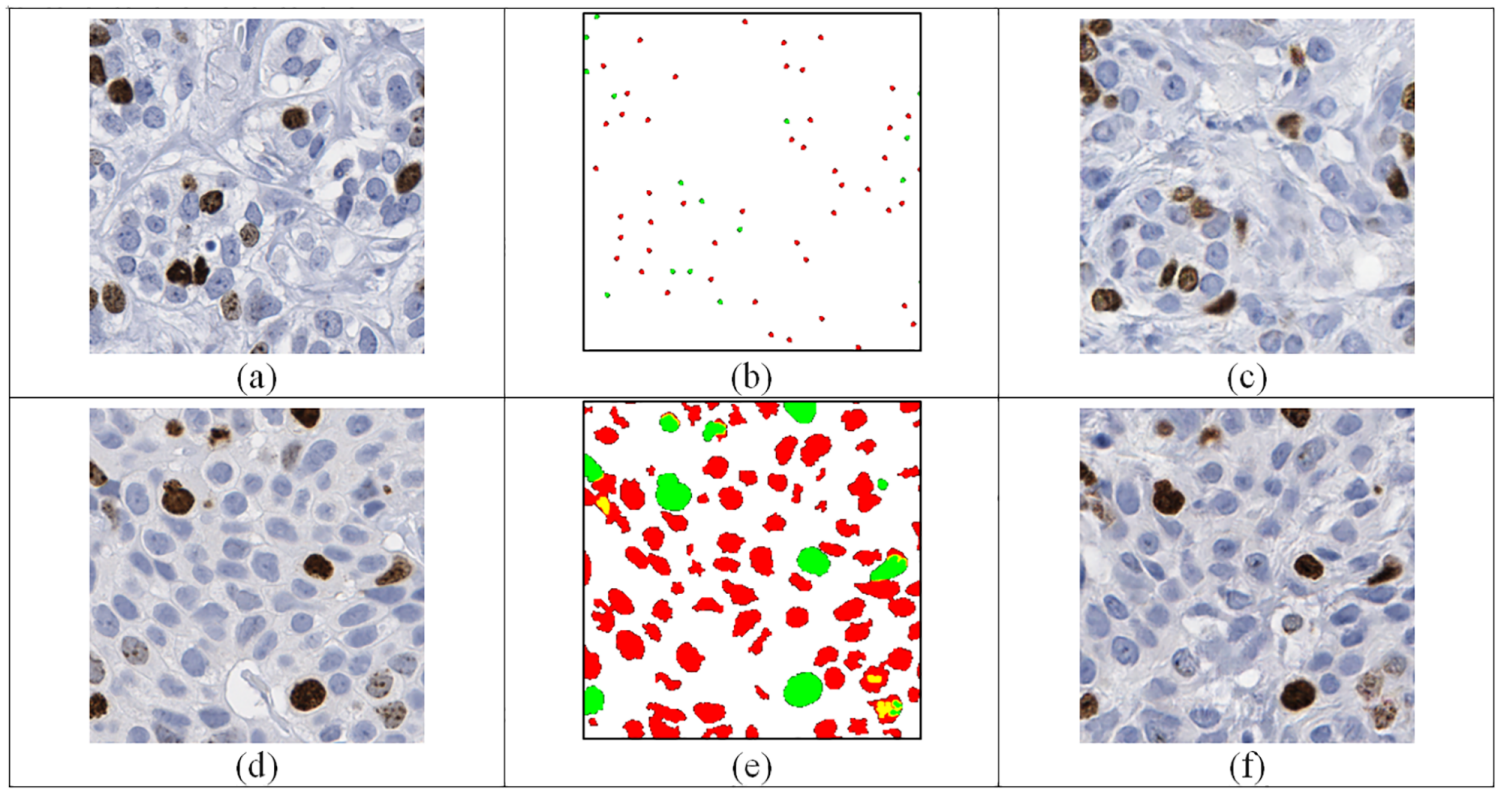
Example images (a) original image used for annotation (b) a dot based annotation, (c) cGAN generated synthetic image from (b). (d) Original image used for segmentation (e) segmentation result using [23], (f) cGAN generated image from (e).

Fig 5 shows some example input images, associated masks (either user annotations or computer segmentations) and generated synthetic images. Fig 5b shows the manual nuclei annotations for an example image in Fig 5a. The output of the generator is given in Fig 5c. Similarly, the output of the generator by using the segmentation algorithm’s output (Fig 5e) is given in Fig 5f. The numbers of correctly identified real and synthetic image by readers are given in Table 1.

**Table 1:** Experts’ discrimination performance on synthetic/real images. TP represents the number of correctly identified synthetic images and TN represents the number of correctly identified real images.

	Pathologist 1			Pathologist 2			Pathologist 3			Image Analyst 1			Image Analyst 2			Image Analyst 3		
	TP	TN	TP+ TN	TP	TN	TP+ TN	TP	TN	TP+ TN	TP	TN	TN+ TP	TP	TN	TN+ TP	TP	TN	TN+ TP
Dataset1 (10 synthetic)	2	0	2	6	0	6	6	0	6	4	0	4	10	0	10	4	0	4
Dataset2 (10 real)	0	6	6	0	6	6	0	4	4	0	5	5	0	1	0	0	6	6
Dataset3 (5 synthetic, 5 real)	0	2	2	4	2	6	2	3	5	2	3	5	3	1	4	3	3	6
Accuracy	33.3%			60.0%			50.0%			46.7%			46.7%			53.3%		

According to our hierarchical logistic regression model, the probability that the average reader would be able to correctly classify an image as synthetic or real more than 50% of the time was only 44.7%. These results suggest that, overall; readers are incapable of distinguishing synthetic images from real ones. However, the results differed by data set: when presented with a data set comprised entirely of real images, the posterior probability of correctly classifying an image more than 50% of the time was 70.4% compared to only a 30.5% probability for data set 1 (100% synthetic images) and a 40.1% probability for data set 3 (50% synthetic, 50% real). The improved classification performance in dataset 1 could be due to a tendency of readers to label images as “real” slightly more often than “synthetic” (54% of the time compared to 46% of the time based on the data for the four readers used in the modeling).

**Fig 6:**
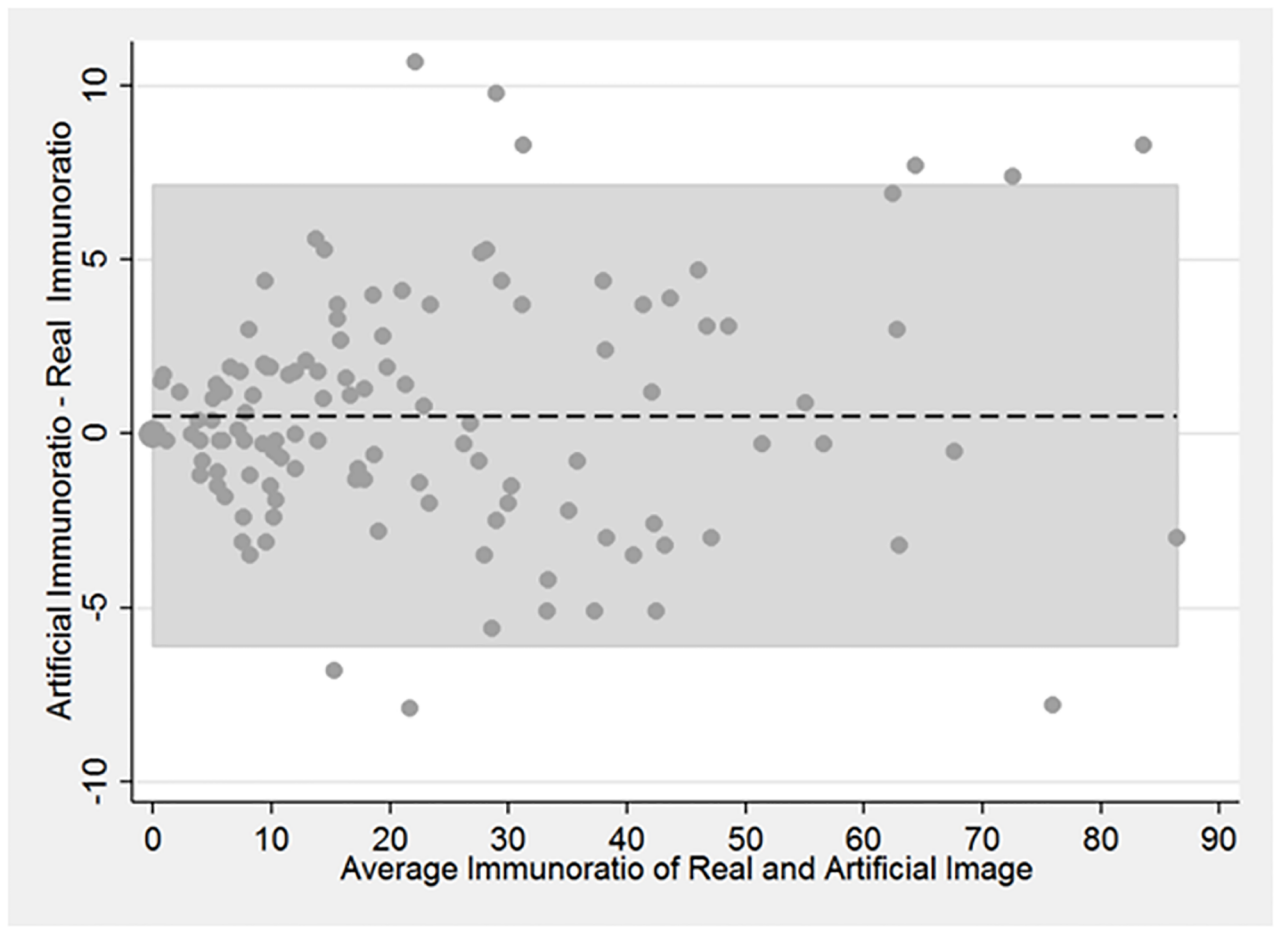
Bland-Altman plot comparing ImmunoRatio values of real and artificial images. Shaded region corresponds to the limits of agreement.

**Fig 7:**
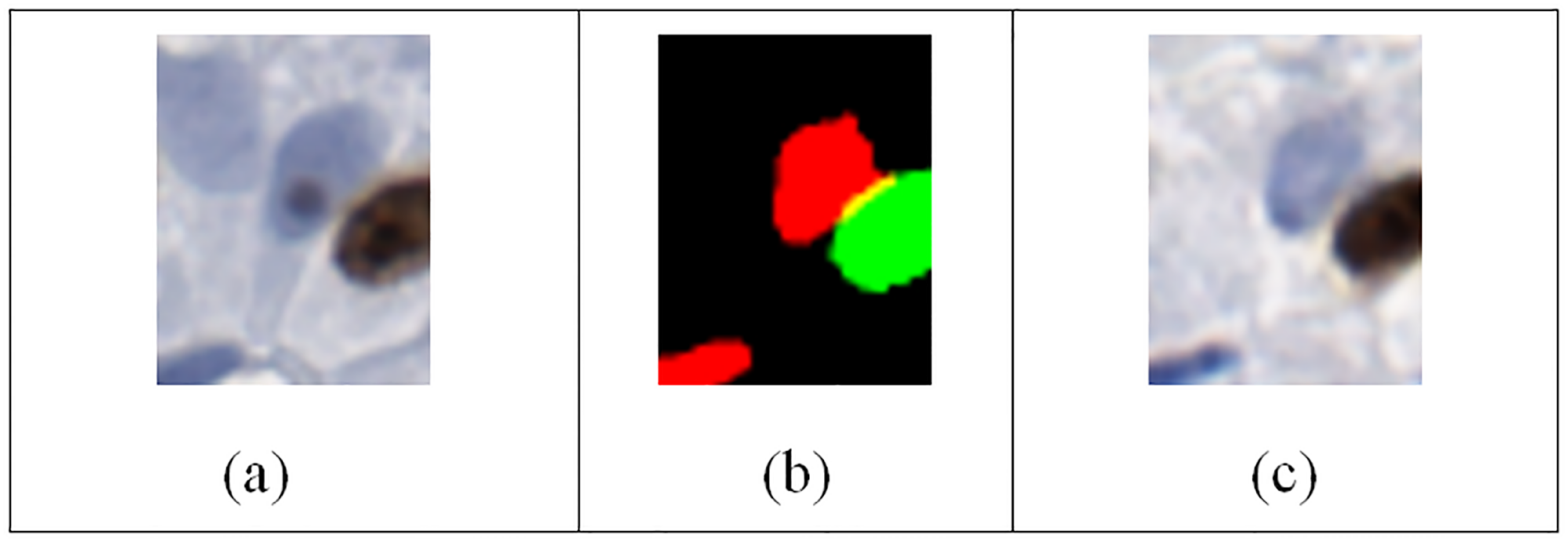
An example case where the immunoRatio values are different in the real and synthetic images. The upper left nucleus in the real image (a) was missed by the segmentation result (b) based on [5]. Therefore the synthetic image (c) was not including that nucleus.

When analyzing the real and synthetic images using computerized quantitative methods, we examined the ImmunoRatio differences between real and synthetic images. Fig 6 displays the Bland-Altman analysis of the ImmunoRatio data. The average difference in ImmunoRatio values was 0.53 and the difference did not appear to depend on value of the ratio. Considering that ImmunoRatio is a percentage (i.e., its value ranges between 0% and 100%), this average difference (estimated bias) is very small. The limits of agreement between the two images were (-6.1, 7.1). Furthermore, the concordance correlation coefficient was 0.99 (95% CI: 0.98–0.99) which indicates almost perfect agreement between the ImmunoRatio values of the real and artificial images. Fig 6 displays the Bland-Altman analysis of the ImmunoRatio data. There were two main reasons for the observed ImmunoRatio differences between the real and synthetic images. The first reason was the initial segmentation algorithm may result in some false positives and false negatives. This error was propagated to the synthetically generated image (Fig 7). Similarly, the ImmunoRatio algorithm may generate some false alarms, and false negatives for both of the images and the amount of error may change depending on the difference of their color characteristics.

## Conclusions

In this study, we proposed a novel method to create realistic synthetic histopathological breast cancer images with Ki67 staining by using conditional Generative Adversarial Networks. The proposed method is different from the prior synthetic tissue generation approaches by producing realistic synthetic images that are hard to distinguish from their real counterparts even by experienced pathologists. For training, two different input methods are evaluated: manual nuclei location annotations and segmentation masks generated by a computer algorithm. We observed that using the segmentation masks provides several advantages over manual annotations. First, it allows defining size, orientation and shape information for each nucleus. Second, unequivocal staining conditions (i.e. a nuclei that cannot be easily labeled as negative or positive) can be simulated with this approach (e.g. yellow color regions in Fig 3b). Finally, using an existing segmentation algorithm suppress the need of manual annotation during the training.

This study has several practical implications. The artificially created datasets with known ground truth can allow researchers to analyze the accuracy, recall, precision, and intra- and inter-observer variability in a systematic manner and compare the human readers with a computer analysis. The algorithm has the potential to generalize to different types of carcinomas (e.g. neuroendocrine, bladder cancers, etc.) and produce an unlimited number of teaching cases for pathology residents. For instance, we can modify the proposed algorithm to produce different levels of invasion in bladder cancer to train pathology residents in staging bladder cancer pathology. In addition, this approach may help algorithm developers for not only evaluating their methods but also for generating unlimited training and testing samples for algorithm development.

This study also has several practical applications. For example, currently, each laboratory within United States uses locally devised tissue slide preparation and scanning protocols. The study is significant as it has the potential to assist in careful selection of technical parameters that directly affect the tissue slide preparation and its display and also assist in regular checking of scanner performance with measurement of physical image parameters. Both, the technical parameters and the physical parameters have the potential to bring standardization to digital slide preparation process. Moreover, the study can assist in devising new standards to compare the quality of different scanners. Finally, it is worth mentioning that the proposed method can be easily generalized to other stains (such as CD3, CD4, CD8, CD21 etc.) and diseases (e.g., lung, colon, prostate cancer, kidney disease, etc.)
